# Defining the real-world reproducibility of visual grading of left ventricular function and visual estimation of left ventricular ejection fraction: impact of image quality, experience and accreditation

**DOI:** 10.1007/s10554-015-0659-1

**Published:** 2015-07-04

**Authors:** Graham D. Cole, Niti M. Dhutia, Matthew J. Shun-Shin, Keith Willson, James Harrison, Claire E. Raphael, Massoud Zolgharni, Jamil Mayet, Darrel P. Francis

**Affiliations:** International Centre for Circulatory Health, National Heart and Lung Institute, Imperial College London, 59-61 North Wharf Road, London, W2 1LA UK; Division of Imaging Sciences and Biomedical Engineering, King’s College London, London, UK; Royal Brompton Hospital, London, UK

**Keywords:** Echocardiography, Ventricular function, Heart failure, Reproducibility of results

## Abstract

Left ventricular function can be evaluated by qualitative grading and by eyeball estimation of ejection fraction (EF). We sought to define the reproducibility of these techniques, and how they are affected by image quality, experience and accreditation. Twenty apical four-chamber echocardiographic cine loops (Online Resource 1–20) of varying image quality and left ventricular function were anonymized and presented to 35 operators. Operators were asked to provide (1) a one-phrase grading of global systolic function (2) an “eyeball” EF estimate and (3) an image quality rating on a 0–100 visual analogue scale. Each observer viewed every loop twice unknowingly, a total of 1400 viewings. When grading LV function into five categories, an operator’s chance of agreement with another operator was 50 % and with themself on blinded re-presentation was 68 %. Blinded eyeball LVEF re-estimates by the same operator had standard deviation (SD) of difference of 7.6 EF units, with the SD across operators averaging 8.3 EF units. Image quality, defined as the average of all operators’ assessments, correlated with EF estimate variability (r = −0.616, *p* < 0.01) and visual grading agreement (r = 0.58, *p* < 0.01). However, operators’ own single quality assessments were not a useful forewarning of their estimate being an outlier, partly because individual quality assessments had poor within-operator reproducibility (SD of difference 17.8). Reproducibility of visual grading of LV function and LVEF estimation is dependent on image quality, but individuals cannot themselves identify when poor image quality is disrupting their LV function estimate. Clinicians should not assume that patients changing in grade or in visually estimated EF have had a genuine clinical change.

**Electronic supplementary material:**

The online version of this article (doi:10.1007/s10554-015-0659-1) contains supplementary material, which is available to authorized users.

## Introduction

Clinicians are sometimes surprised that a patient moves between normal and impaired left ventricular function with just re-assessment of the same acquired images. Outside of research, qualitative grading of ventricular function using portable hardware with limited functionality is common [1, 2]. An alternative is the similarly speedy “eyeball” EF [3], in which the recommended formal Simpson’s calculation [4] is not carried out but a judgment is made from the images alone. It is apparent that this practice occurs not only in clinical practice but also in recruitment for landmark randomized controlled trials. REVERSE [5] and MADIT-CRT [6], for example, have disclosed the histograms of EF values from recruitment centers, which suggest that the majority were eyeball estimates.

Patients undergoing echocardiography for clinical reasons may have images that would not be of the quality typically displayed as published examples [7] of the technique. Whilst previous studies have shown that visual estimation and formal calculation of EF have a strong relationship [8–10], it is not known whether the reproducibility of qualitative grading of LV function and visual estimation of ejection fraction is resilient to imperfect image quality.

The use of bedside echocardiography as an extension of the clinical examination is desirable [2] and increasingly affordable [1]. Improved access makes serial reassessment during the same episode of care possible. This portable hardware often has limited functionality, leaving operators to judge LV function on visual appearance without access to the full panel of measurements.

Current guidelines already discourage short-cut estimation of LV function [4]. Whether these techniques should be universally discouraged for *all* cases regardless of image quality and for *all* operators regardless of experience and accreditation status is unknown.

In this study, in a cohort of patients undergoing routine clinical inpatient or outpatient echocardiography, we defined the reproducibility of qualitative grading and estimation of EF, and quantified the impact of image quality, experience and accreditation.

## Methods

We selected 20 anonymous apical four-chamber echocardiograms acquired using a General Electric Vivid I (General Electric, Hatfield, UK) or Philips ie33 (Philips, Guildford, UK). The cine loops, as seen by operators, are shown in Online Resources 1–20. Two of the authors (GDC, DPF) reviewed the studies to ensure that there was a range of image quality and LV function across the studies. Each echocardiogram was duplicated, so that there was the appearance of 40 studies. The studies were ordered randomly in a Powerpoint presentation and viewed by study participants unaware of the duplication.

We did not impose a time limit for operators, because we wanted to simulate normal practice in which operators would be free to spend as much time as they wished. Operators in this study generally spent less than a minute viewing each case.

Operators were provided with a data entry sheet (Online Resource 21) that asked them to provide:

A. an overall visual grading of LV function (either hyperdynamic, good/normal, mildly impaired, moderately impaired or severely impaired)
B. an “eyeball” estimate of LV function (expressing a range was permitted e.g. 40–50 %)
C. a judgment of image quality by marking on a visual analogue scale running from 0 (worst quality imaginable) to 100 (best quality imaginable)

We also recorded:

1. whether they were accredited by an echocardiographic society
2. the operators number of years of experience in echocardiography

Where an EF range (such as 20–30 %) was given, the midpoint of the range was taken as the value (such as 25 %). Statistical analysis was undertaken using “The R project for statistical computing” [11] with Figures prepared using “ggplot2” [12]. Normal distributions were expressed as mean and standard deviation and tested with Pearson’s product moment correlation and *t* test. We undertook a linear regression of image quality, experience and accreditation status against distance of estimates from the consensus of all operators.

## Results

### Cases

The average age of patients was 60.7 ± 15.8 years. 11 (55 %) were male and 9 (45 %) were female. The indications for echocardiography were to assess: LV function (7, 35 %), valvular function (4, 20 %), cause of stroke (3, 15 %), LVH (2, 10 %), RV function (2, 10 %), regional wall motion abnormalities (1, 5 %) or cause of palpitations (1, 5 %). The cases, as seen by operators, are shown in Online Resources 1–20.

### Operator characteristics

35 operators from three institutions reviewed the cases. Their median experience of echocardiography was 4 years (interquartile range 2–6 years). 19 (54 %) held formal accreditation.

### Visual grading of LV function

35 operators reviewed 20 videos twice, creating 700 possible paired assessments of the same echocardiogram. There were 42 blank responses, 10 responses of “can’t grade” and 35 responses that were not a single grading, for example “moderate to severe”. These 87 ineligible responses affected 63 pairs, leaving 637 paired assessments for intra-operator analysis.

### Reproducibility of visual grading of a cine loop by the same operator

**Fig. 1:**
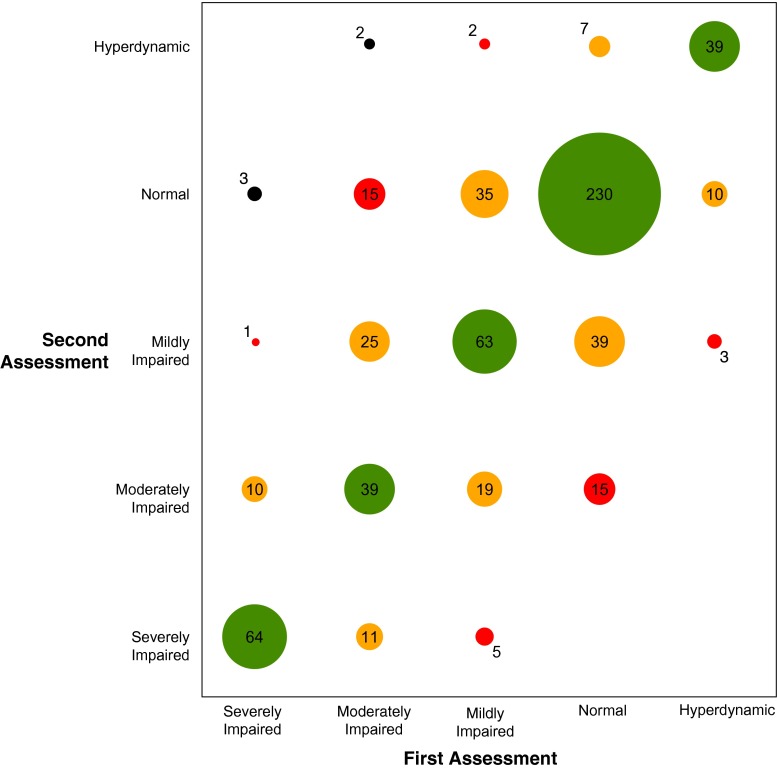
Intra-operator self-disagreement by operators reassessing the same images blind to their previous assessment. The *area of the bubbles* represents the frequency of assessments with this combination

Overall, 435(68 %) of the 637 videos that were assessed and then re-assessed were given the same visual grading when represented to the same operator (shown as green bubbles in Fig. 1). In 156(24 %), the gradings by the same operator viewing the same images differed by one category (orange bubbles). In 41(6 %) the gradings by the same operator viewing the same images differed by two categories (red bubbles). In 5(1 %), the gradings by the same operator viewing the same images differed by three categories (black bubbles). No pairs differed by four categories. Online Resource 22 shows Fig. 1 with responses classified as to whether operators were accredited (and typically more experienced, left panel) or non-accredited (and typically less experienced, right panel). The pattern of intra-operator disagreement is very similar, although there is a tendency for non-accredited operators to disagree with themselves by many categories more frequently than accredited operators.

Although disagreement by the same observer on representation was common (32 %), disagreement by more than one category was uncommon (7 %). However, even disagreement by one category (24 %) may be important, if it is informing the decision about whether cardiac function is normal (in which further tests are unlikely) or abnormal (in which further tests may be undertaken). When the data are analysed by dichotomizing visual gradings into normal (including hyperdynamic) versus abnormal (impairment of any severity), the same operator viewing the same images came to the same dichotomous decision in only 523 (82 %) of cases.

### Reproducibility of grading of cine loop across different operators

**Fig. 2:**
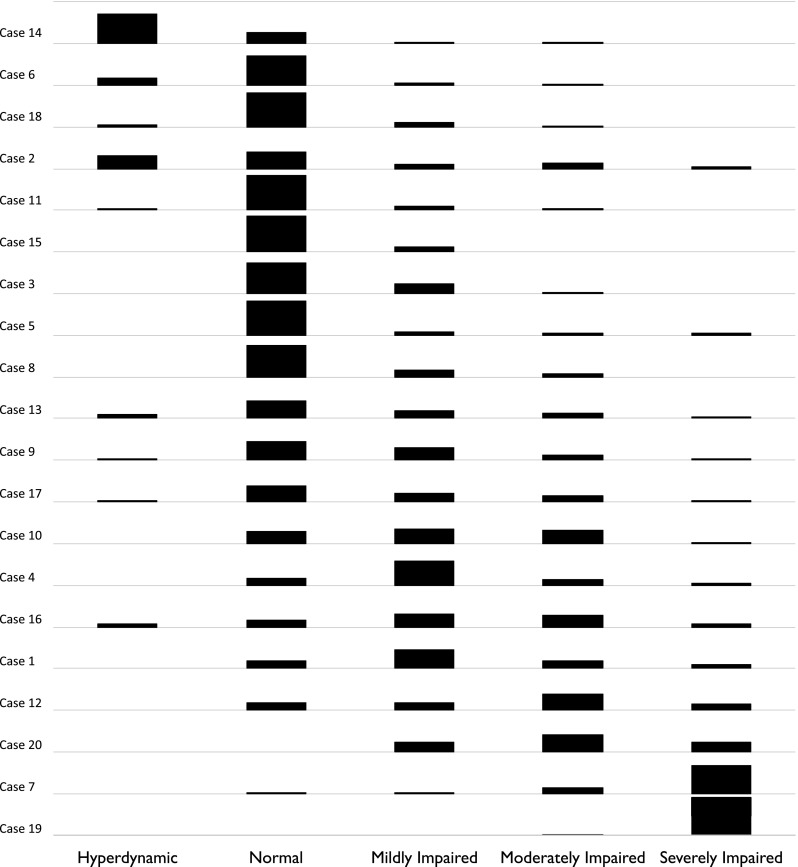
Inter-operator disagreement by different operators reassessing the same images. Each of the 20 rows is a different case. The *row* shows a histogram of the 70 assessments made by the 35 operators for that case. In this figure, cases are ordered by the average grading given by operators

There were five available categories for visual grading. Only one case was visually graded the same by all operators on one set of viewings (but when the images were re-presented this did not hold), as shown in Fig. 2. In 6/40 cases (15 %) two of the five grades were used. In 9/40 (23 %) three grades were used. In 17/40 (43 %) four grades were used. In 7/40 (18 %) all five grades were used. Across all cases, the chance of agreeing with another operator was 49 % on the first viewing and 52 % on the second viewing. Agreement can be seen to be relatively good at the extremes, but less so in the intermediate region, including differentiating normal from mild or moderately impaired.

When responses were dichotomized into normal (including hyperdynamic) and abnormal (impairment of any severity), the chances of a given operator agreeing with another that LV function was normal or abnormal was 70 % on the first viewing and 73 % on the second viewing.

### Estimation of ejection fraction

35 operators reviewed 20 videos twice, creating 700 possible paired estimates of ejection fraction. There were 59 blank responses and 1 “can’t grade” response affecting 39 pairs, leaving 661 pairs of estimates. The average EF estimate given by all operators for all cases was 50.1 EF units ±13.5 units.

### Reproducibility of reading a cine loop by the same operator

**Fig. 3:**
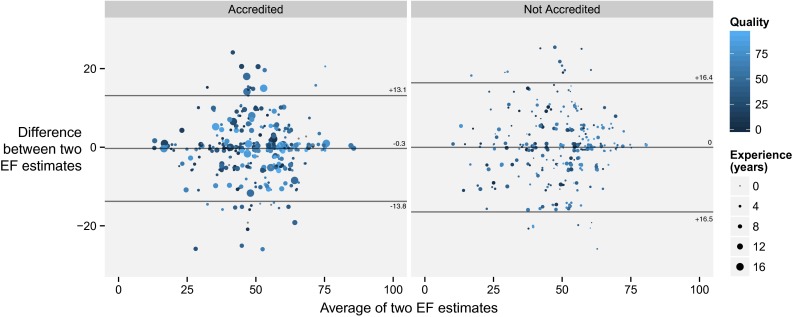
Bland-Altman plots of first and second EF estimates when the same case was re-presented to the same operator. The *left panel* shows accredited operators. The *right panel* shows non-accredited operators. *Paler blue* points are an estimate where the operator judged the image of high quality, whereas *dark blue* represents a poor quality image. *Larger dots* indicate more experienced operators. In drawing this graph we have added a random ±1 % “jitter” so that multiple identical values may be appreciated

The standard deviation of the difference between first and second EF estimates for all cases by all operators was 7.6 LVEF units (Fig. 3).

### Reproducibility of estimating EF for a cine loop across different operators

**Fig. 4:**
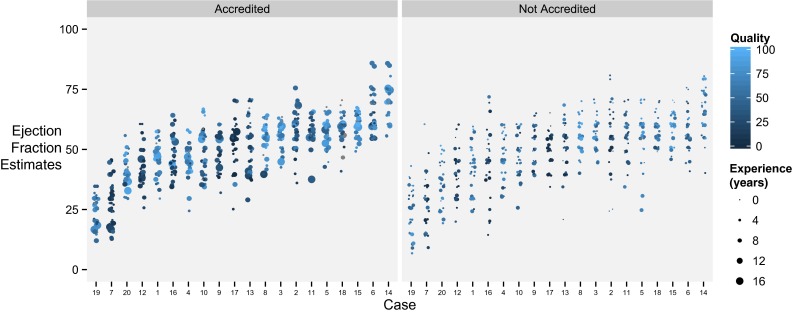
Visual estimation of EF for 20 different cases, arranged from lowest average EF to highest average EF. *Each column* represents one echocardiographic case ordered from lowest average EF to highest average EF. The *points* in the column represent the EF estimates by 35 operators viewing the images twice.* Paler blue* points are an estimate where the operator judged the image of high quality, whereas *dark blue* represents a poor quality image. *Larger dots* indicate more experienced operators. In drawing this graph we have added a random ±1 % “jitter” so that multiple identical values may be appreciated

The standard deviation of EF estimates by all operators for a given case averaged 8.3 LVEF units ±1.7 LVEF units. The individual estimates are shown in Fig. 4.

### Quality assessment

35 operators reviewed 20 videos twice, creating 700 possible paired assessments of image quality where the same operator views the same echocardiogram. One operator failed to provide any quality assessments. Across the other operators, there were 20 blank responses affecting 18 different pairs. In total, this left 662 paired assessments of the 20 cases. Some operators chose to write a number rather than draw on the Likert diagram provided: this was accepted. The average quality assessment given by all operators for all cases was 49.0 ± 27.3.

### Reproducibility of image quality rating by the same operator

**Fig. 5:**
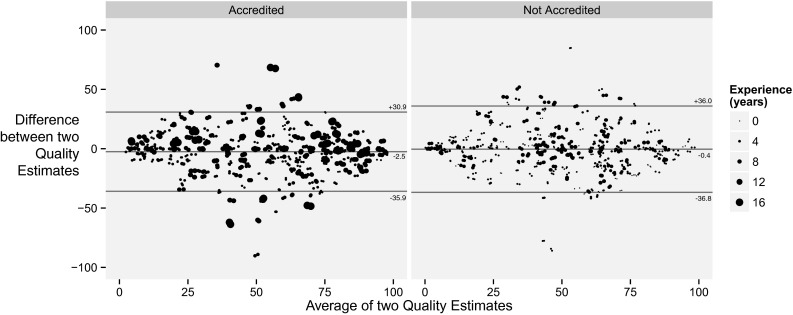
Bland-Altman plot of intra-operator self-disagreement by operators reassessing the quality of the same images blind to their previous assessment. The *left panel* shows accredited operators. The *right panel* shows non-accredited operators. *Larger dots* indicate more experienced operators. In drawing this graph we have added a random ±1 “jitter” so that multiple identical values may be appreciated

The standard deviation of the difference between each operator’s first and second assessment was 17.8, as shown in Fig. 5.

### Reproducibility of image quality rating by different operators

**Fig. 6:**
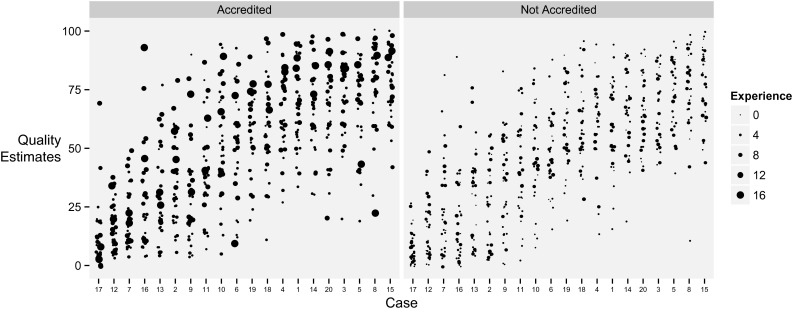
Inter-operator disagreement by operators assessing quality for the same images. *Each column* represents one echocardiographic case ordered from lowest average quality to highest average quality. The *points* in the column represent the assessment of quality by 35 operators viewing the images twice. Quality was assessed on a 0–100 scale. The cases are arranged from lowest to highest mean quality score. In drawing this graph we have added a random ±1 “jitter” so that multiple identical values may be appreciated

The standard deviation of quality estimates by all operators for a given case averaged 17.4 ± 2.8. The individual estimates are shown in Fig. 6.

### The effect of image quality on reproducibility (as assessed by all operators)

We defined the image quality of a case as the mean of both quality assessments made by all operators for that case.

### Visual grading and image quality

**Fig. 7:**
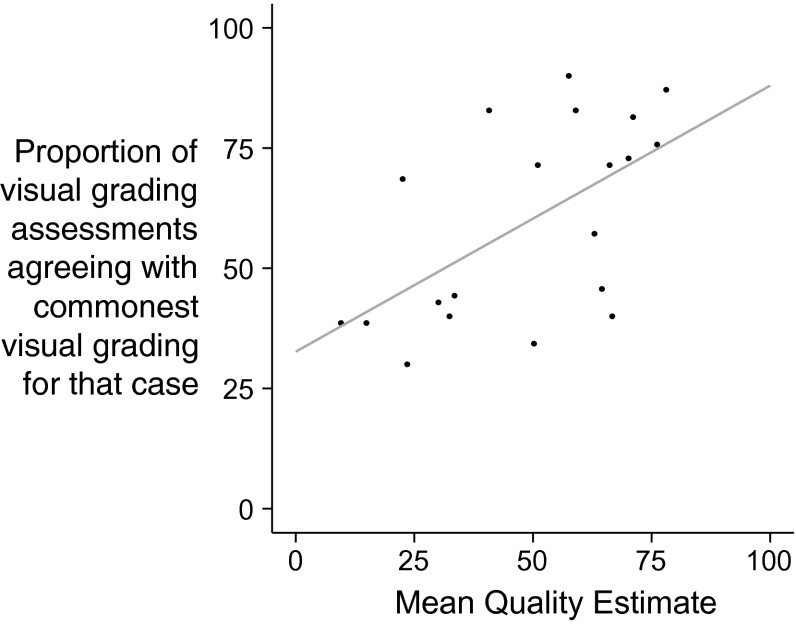
Better image quality allows observers to agree with each other on ventricular function. *Each point* represents the proportion of visual grading assessments that agreed with commonest function assessment for that case versus the average quality score awarded by all observations of that case

Image quality was correlated with the agreement of different operators, as assessed by the proportion of assessments in the modal category (Pearson r = 0.58, *p* < 0.01), Fig. 7.

### EF estimation and image quality

**Fig. 8:**
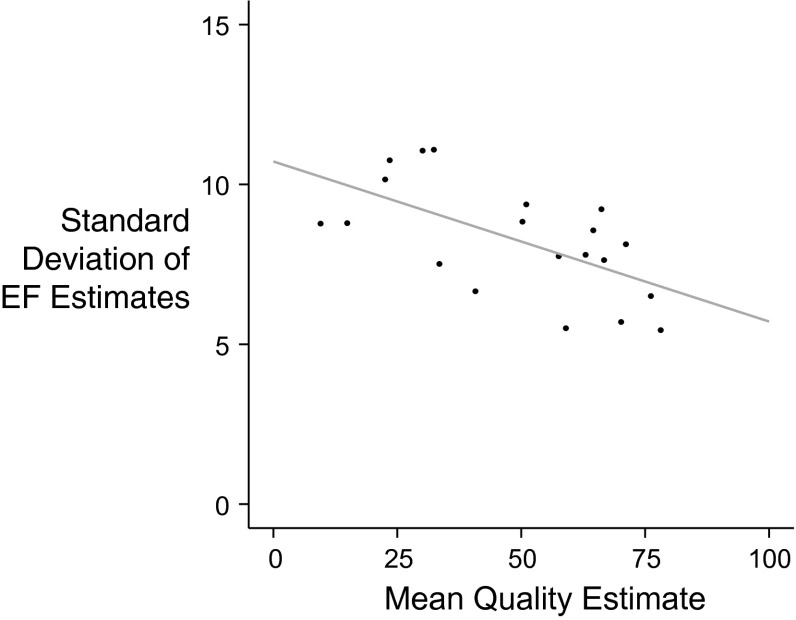
Impact of image quality on variability of EF estimates. *Each point* represents the standard deviation of estimates between operators for a case versus the mean quality estimate by all operators for that case

As shown in Fig. 8, the agreement of EF estimates improved with better image quality. The standard deviation of all EF estimates for all operators viewing a case correlated inversely with image quality for that case (r = −0.616, *p* < 0.01). Despite the improvement with image quality, even the cases with the best quality images have a standard deviation of EF estimates between observers of at least 5 EF units.

### Inability of individuals to identify when they are providing an outlying visual grading or LVEF estimate

Although reduced variability in visual grading and LVEF estimates did correlate with the group’s consensus of image quality, an individual observer judging whether his or her own assessment of ventricular function is likely to be reliable in clinical practice has access to only his or her own personal estimate of image quality.

We therefore considered whether individuals assessing a particular case as having lower image quality were more likely to have provided an outlying visual grading or LVEF assessment for that case.

**Fig. 9:**
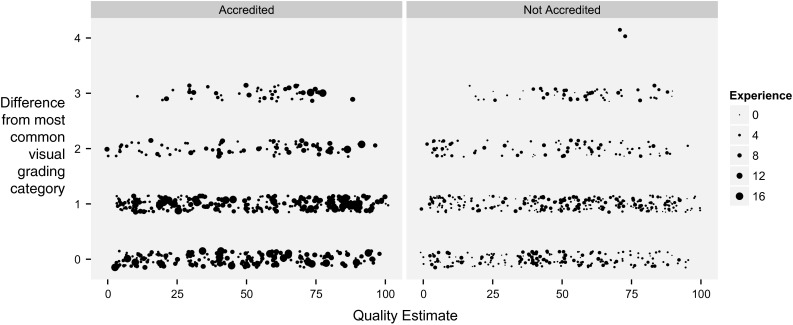
Relationship between quality score and the number of categories’ deviation from the modal consensus of visual grading. *Each point* represents how many categories a single operator’s visual grading is from the modal visual grading versus the quality assessment the individual operator made at that time. The *left panel* shows accredited operators. The *right panel* shows non-accredited operators. *Larger dots* indicate more experienced operators

As shown in Fig. 9, there was little useful relationship between an individual operator reporting a low image quality score and them providing an outlying visual grading. Although there was a significant fall in distance from the mode with improved image quality (*p* < 0.01), agreement improved so modestly with improved image quality that an image assessed as the best possible quality is likely to be only 0.3 categories closer to the modal category than one with the worst possible quality. The result is that there is no useful cut-off of image quality beyond which an individual operator can predict when they are making an outlying visual grading.

**Fig. 10:**
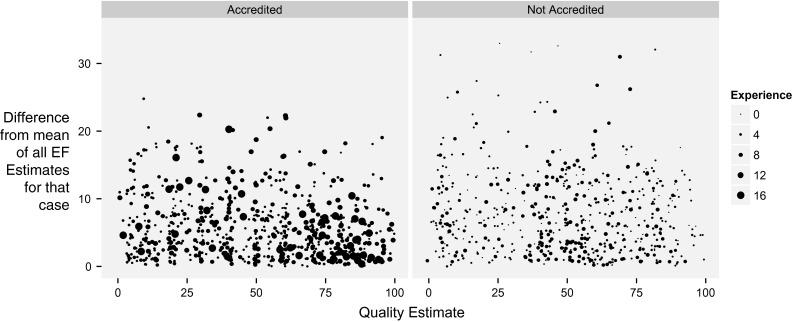
Relationship between quality score given by an operator and the difference in their EF estimate from the mean of all operators. *Each point* represents the absolute difference a single operator’s EF estimate is from the mean of all operators versus the quality assessment the individual operator made at that time. The *left panel* shows accredited operators. The *right panel* shows non-accredited operators.* Larger dots* indicate more experienced operators

As shown in Fig. 10, there was similarly little useful relationship between an individual operator reporting a low image quality score and them providing an outlying visual estimate of EF. Although there was a significant fall in distance from the mean with improved image quality (*p* < 0.01), agreement improved modestly with improved image quality, so that an image assessed as the best possible quality is likely to have an EF estimate 2.5 EF units closer to the mean than one with the worst possible quality. The result is that there is no useful cut-off of image quality beyond which an individual operator can predict when they are making an outlying visual estimate of EF.

### The effect of experience and accreditation

#### Visual grading

Experience is represented by the size of dots on Fig. 9. Increasing experience did not reduce the number of categories deviation from the modal consensus of visual gradings for that case (r = −0.01, *p* = n.s.). Accredited operators (left panel of Fig. 9) provided visual gradings 0.95 ± 0.87 categories from the consensus compared with 1.04 ± 0.94 for non-accredited operators (right panel of Fig. 9), *p* = n.s.

#### Estimation of EF

Experience is represented by the size of dots on Fig. 10. Increasing experience did not reduce the distance of EF estimates from the mean for that case (r = 0.02, *p* = n.s.). Accredited operators (left panel of Fig. 10) provided estimates of EF that were significantly closer (by 1.55 EF units, *p* < 0.01) to the mean for that case than non-accredited operators.

## Discussion

Visual grading and eyeball estimation of ejection fraction, widely used in clinical practice and in research, can lead to widely variable assessments between operators and even within the same operator. This occurs even when looking at identical images, i.e. with contributions from biological variability and acquisition technique removed.

### Pocket-sized cardiac ultrasound

The challenge of reproducibly assessing LV function exists for all imaging modalities, but is pertinent to bedside echocardiography for two reasons.

Firstly, it is often the first modality used to assess LV function. The European Association of Echocardiography is wisely cautious because of the lack of quantification on many portable devices, but its position statement [13] suggests that pocket-sized ultrasound devices might help the triage of candidates for a complete echocardiographic examination. If the pocket-sized assessment is rated “normal”, the patient might therefore not undergo a full examination. In this study, when the same operator viewed the same image again only minutes apart, almost 1 in 5 visual gradings were changed from “normal” to impairment of some severity or the reverse, indicating that, even as a triage technique, we should be cautious.

Secondly, the portability, affordability and lack of ionizing radiation mean that portable echocardiography devices might come into use for serial reassessment of LV function for hospital inpatients. Defining and improving the reproducibility of assessments is essential if we are to detect genuine clinical changes amongst noise.

### Is agreement really better at extremes?

Agreement between operators visually grading LV function (Fig. 2) appears more likely if the case is at the extremes of LV function. This mirrors our own experience that we find it easier to agree when cases are either very abnormal or very normal. Another explanation is that agreement occurs at extremes because the limited range of responses available masks the normal variation from multiple assessments that arises for less extreme cases. For example in Case 19 (bottom row of Fig. 2), almost all operators agree that the LV function is severely impaired, but if a further category was available (e.g. *super*-severely impaired), some of the responses might be distributed into the further category, reducing the calculated level of agreement. In support of this, we saw no evidence of better agreement in EF estimation where the average EF was either very high or very low (Fig. 4), presumably because none of our cases (mean EF 24–68 %) were close enough to 0 or 100 % for those numerical limits to restrict choice and therefore cause bunching of answers.

### Is an operator’s perception of image quality a safe pointer to reliable estimation of function?

One contributor to variability in visual grading and LVEF estimates is indeed image quality. We found a statistically significant tendency for images judged by the group as poor quality to have a wider variability between observers in the judgment of ventricular function. However, this study provides additional insights into this process.

Firstly, we found that individuals do not agree with each other on image quality. Since the disagreement on image quality within individual observers is large, this is not because different individuals disagree; rather the task is inherently (and deceptively) difficult. When image quality assessments are crowd-sourced across many observers, cases with better quality show better agreement between observers regarding ventricular function.

However, although individuals’ estimates are closer to the consensus when they rate an image as high quality, the degree of improvement as quality improves is modest. No cut-off can help a single observer to use self-perceived image quality as an effective predictor of whether their opinion of LV function will match those of other observers or not.

In practical terms this means that, unfortunately, an observer judging an image to be of good quality should not feel secure that this means that other observers would agree with their judgment on ventricular function grading or ejection fraction.

Future development of automated algorithms for assessing imaging quality may be useful to resolve this. However it would be advisable to use as a reference standard the opinion of not just one observer but a panel of observers. The panel members should also be mutually blinded to permit them to contribute genuinely independent information into the pool.

Even if there was a reliable index of image quality, however, the trend to improved reproducibility of ejection fraction with improved image quality is sufficiently weak that, even in the highest quality images, the variation between observers in ejection fraction had a standard deviation of ~5 % points, i.e. a 95 % confidence interval that is ~20 % points wide.

### Previous studies

A number of previous studies demonstrate high correlation between visual estimation of ejection fraction and other techniques such as radionuclide ventriculography [8–10]. The guideline [4] is much more cautious, a position which our data supports. It is unclear how visual estimation can correlate so well with other techniques in other studies when we have found it correlates poorly with itself, but our study included a much larger number of operators than previous studies and asked them to study a clinically realistic wide range of image quality.

When a technique is reported to be less reliable than hoped [14, 15], it is tempting for us to assume that this is because it has been carried out inexpertly [16, 17]. An alternative explanation is that the technique may appear reliable in the hands of unblinded experts demonstrating cases agreed by all to be exemplary, but falls short when an unselected patient cohort is examined under bias-resistant conditions, even with experienced operators. In this study all participants used echocardiography regularly and had no reason to deliberately underperform.

When weighing up why similar studies can produce a spectrum of different results, we believe it is much easier for interested readers if they have access to the raw data to permit re-analysis [18]. In the past, providing imaging data used in our analysis [19] has allowed queries [20] to be resolved productively [21]. We have therefore provided our data with operators made non-identifiable as Online Resource 23. In addition, we show the videos of all cases as Online Resources 1–20 so that readers can appreciate that the cases showed encompassed a real-world spectrum of image quality. We hope to encourage future work adopting a similar open approach.

### Importance of training

Accreditation improved agreement on LV function assessment, consistent with the findings of Johri et al. [22] who have demonstrated improvement following a teaching intervention. However, very few operators were able to place more than three quarters of the cases into the category selected by most operators. Similarly, the reproducibility of LVEF estimates improved only weakly: the standard deviation of difference for individual operators is rarely <5 EF units. Our interpretation is that there is a “ceiling” of reproducibility inherent to visual grading and estimation, and that it may be unreasonable to expect performance better than this ceiling even with experience and accreditation.

### Limitations of this study

We used only the four-chamber view because it is a common view used when clinicians judge, or display ventricular function to colleagues. In clinical practice, more views are used. However, this study was designed to maximise the chance that the operators would agree. If there were multiple views, different observers might have placed differential emphasis on different views and thereby shown even greater disagreement. The study therefore ensured all operators viewed the same view, so there was no variation from differential emphasis, and the same recorded loop, so there was no variation from any other source.

This is not a study of test–retest reproducibility. This is only repeated viewing of an identical video loop. Test–retest reproducibility must be wider than the variability shown here, as this re-interpretation variability is inevitably present when two different video loops are examined (even if acquired by an unvarying operator).

Our study did not use ventricular contrast. Firstly, contrast is not universally used in point-of-care echocardiography, which is the situation where visual grading of left ventricular function and estimation of ejection fraction is most common. The settings in which eyeball estimation and visual gradings predominate, especially for serial assessments, are not those in which contrast is currently used most avidly.

We did not advise operators to spend a particular time viewing each case because we wanted to simulate normal practice. It is possible that spending more time might improve reproducibility. It is also possible that additional time spent making formal measurements might improve reproducibility, but, as of yet, it is unclear which measurements might provide optimal return on further time investment.

Our operators had a predilection for multiples of five ejection fraction units. However, this preference is shared widely. For example, the great majority in MADIT-CRT appeared to have been enrolled by an eyeball assessment of ejection fraction as candidly reported by MADIT-CRT authors [6]. For this reason we did nothing to prevent observers from following their normal practice when estimating EF.

## Conclusions

There is growing availability of affordable portable cardiac ultrasound hardware [1, 2] which lacks a facility for Doppler, tissue Doppler, or area quantification. Visual grading and “eyeball” EF may therefore appear to be a pragmatic choice for rapid assessment of LV function and charting progress. In this study, a broad spectrum of 35 operators examined 20 real-world video loops twice, providing a representative insight into realistic expectations of agreement between and within operators.

In clinical *practice*, referrers should not assume that a change in visually graded LV function or “eyeball” EF, even if large, indicates a genuine change in their patient’s status. We should also avoid criticizing colleagues who provide different estimates, since this appears to be an unavoidable characteristic of visual estimation.

In clinical *research*, we need to recognize the caveats of these biomarkers. It may be very reasonable to recruit into a trial using a biomarker with poor reproducibility if other attributes (low cost, speed, accessibility) are favourable and the trialled intervention is expected to be effective across the broad patient group. However, if doing so, we should be ready for conflict between observers. We should also recognize that since visual estimates differ so widely from each other, it is certain that any later core lab reassessment will differ from the original visual estimate.

Current guidelines [4] already advise caution in visual estimation of left ventricular function. Our study shows these concerns to be well-grounded. Even usage in triage [13] to a full departmental study should not be assumed to be a secure strategy. Effective clinical practice and research requires us to be aware of the properties of the techniques we use, clearly separating them from inferences regarding personal skill. Identifying, quantifying and discussing sources of variability is a crucial early step.

## Electronic supplementary material

Supplementary material 1 (MOV 5441 kb)

Supplementary material 2 (MOV 6210 kb)

Supplementary material 3 (MOV 4468 kb)

Supplementary material 4 (MOV 5409 kb)

Supplementary material 5 (MOV 4324 kb)

Supplementary material 6 (MOV 3734 kb)

Supplementary material 7 (MOV 4992 kb)

Supplementary material 8 (MOV 9830 kb)

Supplementary material 9 (MOV 6435 kb)

Supplementary material 10 (MOV 7240 kb)

Supplementary material 11 (MOV 7133 kb)

Supplementary material 12 (MOV 8710 kb)

Supplementary material 13 (MOV 5375 kb)

Supplementary material 14 (MOV 9808 kb)

Supplementary material 15 (MOV 5239 kb)

Supplementary material 16 (MOV 7742 kb)

Supplementary material 17 (MOV 5863 kb)

Supplementary material 18 (MOV 7039 kb)

Supplementary material 19 (MOV 6437 kb)

Supplementary material 20 (MOV 6596 kb)

Supplementary material 21 (PDF 184 kb)

Supplementary material 22 (PDF 64 kb)

Supplementary material 23 (XLS 138 kb)

Supplementary material 24 (DOCX 15 kb)
