# Supplementary material for: Defining the real-world reproducibility of visual grading of left ventricular function and visual estimation of left ventricular ejection fraction: impact of image quality, experience and accreditation
Source: Int J Cardiovasc Imaging. 2015 Jul 4;31(7):1303–14. doi: 10.1007/s10554-015-0659-1 (PMC4572050; doi:10.1007/s10554-015-0659-1)
Supplement: Supplementary file 21 — Supplementary material 21 (PDF 184 kb) [file 10554_2015_659_MOESM21_ESM.pdf]

Online Resource 21

Case	LV Function (Hyperdynamic/Normal/Mild/Mod/Sev)	Eyeball EF (Range allowed)	Image Quality 0% 50% 100%
1			
2			
3			
4			
5			
6			
7			
8			
9			
10			
11			
12			
13			
14			
15			
16			
17			
18			
19			
20			

Case	LV Function (Hyperdynamic/Normal/Mild/Mod/Sev)	Eyeball EF (Range allowed)	Image Quality 0% 50% 100%
21			
22			
23			
24			
25			
26			
27			
28			
29			
30			
31			
32			
33			
34			
35			
36			
37			
38			
39			
40			
