# Supplementary figures and images for: Defining the real-world reproducibility of visual grading of left ventricular function and visual estimation of left ventricular ejection fraction: impact of image quality, experience and accreditation
Source: Int J Cardiovasc Imaging. 2015 Jul 4;31(7):1303–14. doi: 10.1007/s10554-015-0659-1 (PMC4572050; doi:10.1007/s10554-015-0659-1)

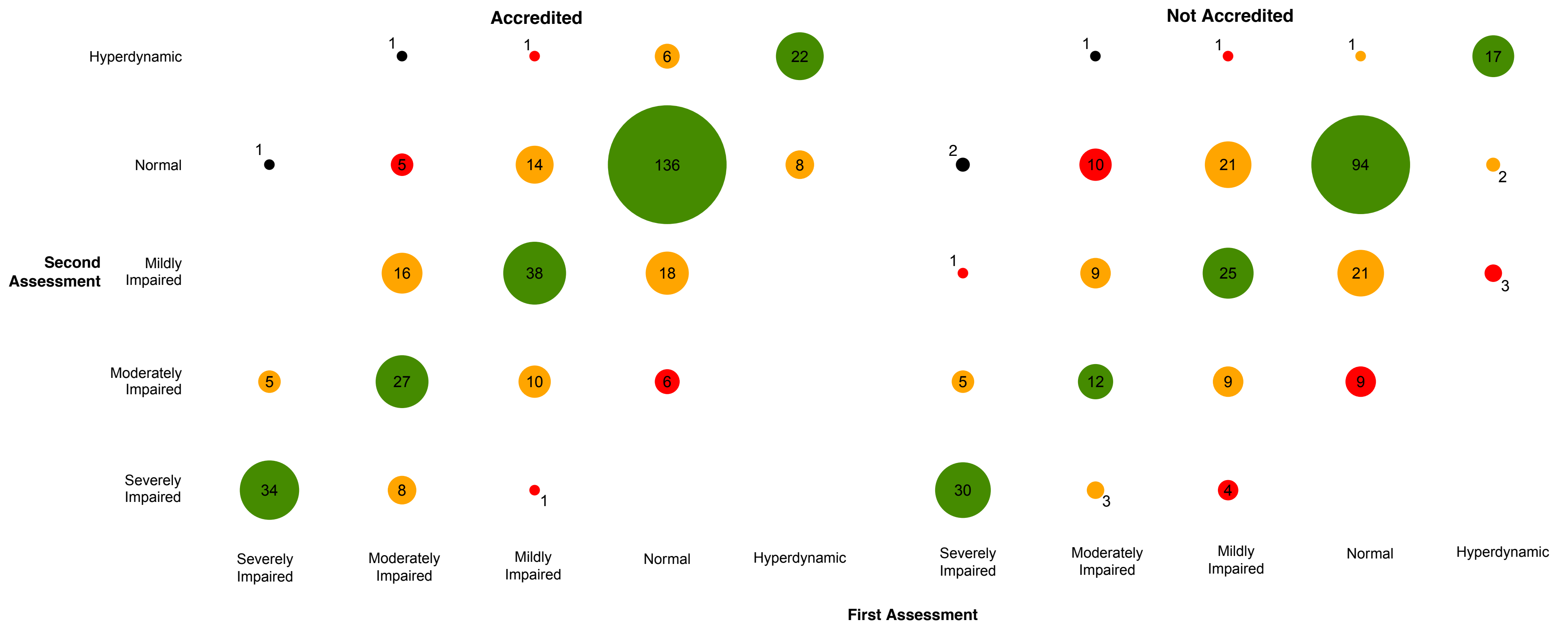

Supplement: Supplementary file 22 — Supplementary material 22 (PDF 64 kb) [file 10554_2015_659_MOESM22_ESM.pdf]
