# Supplementary material for: Defining the real-world reproducibility of visual grading of left ventricular function and visual estimation of left ventricular ejection fraction: impact of image quality, experience and accreditation
Source: Int J Cardiovasc Imaging. 2015 Jul 4;31(7):1303–14. doi: 10.1007/s10554-015-0659-1 (PMC4572050; doi:10.1007/s10554-015-0659-1)
Supplement: Supplementary file 24 — Supplementary material 24 (DOCX 15 kb) [file 10554_2015_659_MOESM24_ESM.docx]

Online Resources

**Online Resources 1 to 20**

Apical four chamber cine loops for cases 1 to 20, as seen by operators taking part in this study.

**Online Resource 21**

Data entry sheet used by participants.

**Online Resource 22**

Intra-operator disagreement in visual grading of left ventricular function. On the left panel are accredited operators (median experience 5 years, interquartile range 3.5 to 8 years). On the right panel are non-accredited operators (median experience 2.5 years, interquartile range 2 to 5.25 years).

**Online Resource 23**

Data collected in this study (with participant experience and accreditation status to ensure participants remain non-identifiable).
